# Effects of trimetazidine on ventricular remodeling in coronary artery disease patients with left ventricular hypertrophy: the rationale and design of a randomized controlled trial

**DOI:** 10.1186/s12872-020-01557-3

**Published:** 2020-06-05

**Authors:** Lili Wu, Yi Luan, Ya Li, Min Wang, Jialin He, Chongying Jin, Wenbin Zhang

**Affiliations:** Department of Cardiology, Key Laboratory of Cardiovascular Intervention and Regenerative Medicine of Zhejiang Province, Sir Run Run Shaw Hospital, School of Medicine, Zhejiang University, 3 East Qingchun Road, Hangzhou Zhejiang, 310016 P.R. China

**Keywords:** Trimetazidine, Ventricular remodeling, Myocardial ischemia

## Abstract

**Background:**

Trimetazidine is a metabolic anti-ischemic agent, which increases the tolerance of cardiomyocytes to ischemia. However, few studies have explored the effect of trimetazidine on ventricular remodeling in coronary artery disease (CAD) patients undergoing percutaneous coronary intervention (PCI) with left ventricular hypertrophy (LVH).

**Methods:**

It is a randomized, placebo-controlled trial, and we propose to recruit one hundred and twenty-four CAD patients undergoing PCI with LVH during a 12-month period. They will be randomized to receive either trimetazidine (35 mg twice a day) or placebo in the following 12 months after PCI. Blood tests, echocardiography, symptom of angina and major adverse cardiovascular events (MACEs) will be collected at follow-up visit at 3 and 12 months. The primary end point will be the left ventricular remodeling measured by left ventricular mass index (LVMI) at 3- and 12-month follow-up compared with the baseline. The secondary end points will be the symptom of angina assessed by Seattle Angina Questionnaire, myocardial ischemia measured by 6-min walk test and exercise electrocardiography test, as well as MACEs (defined as a composite of death, myocardial infarction, stroke, recurrent angina, re-hospitalization, change of viable myocardium).

**Discussion:**

This study aims to demonstrate the effect of trimetazidine on left ventricular remodeling and myocardial ischemia in CAD patients undergoing PCI with LVH. Trimetazidine treatment is likely to improve the left ventricular remodeling, symptoms of angina and myocardial ischemia. It might also reduce the risk of MACEs in CAD patients undergoing PCI with LVH.

**Trial registration:**

http://www.chictr.org.cn, Chinese Clinical Trial Registry (ChiCTR1800017876). Registered on 19 Aug 2018.

## Background

Coronary artery disease (CAD) has become the second leading cause of death after malignant tumor in China, with the prevalence of 6.46% [1, 2]. Left ventricular hypertrophy (LVH), an increase in muscle mass of left ventricle, has been identified as a powerful risk factor for future cardiovascular morbidity and mortality, with the risk of cardiovascular events increasing six-fold to eight-fold [3]. In addition, LVH is independently associated with a higher risk of all-cause mortality and a higher incidence of heart failure in CAD patients treated with percutaneous coronary intervention (PCI) [4–6]. LVH can develop myocytes death and fibrotic remodeling, and this promotes cardiac dysfunction [7], leading to cardiac hypoxia [8].

Besides, microvascular dysfunction is a recognized feature of hypertrophic cardiomyopathy and it may exacerbate myocardial ischemia in CAD patients [9]. Although PCI has become one of the leading treatment methods for coronary heart diseases, microcirculation disturbance and cardiac metabolic disorders caused by LVH still cannot be effectively relieved. Distinctive from other anti-anginal agents, trimetazidine is an anti-ischemic metabolic agent [10–12]. Trimetazidine optimizes the energy utilization of myocardial cells and maintains a proper energy supply during ischemia [13]. It protects the heart by inhibiting oxidative stress [14], scavenging oxygen free radicals [15, 16], improving lipid metabolism and mitochondrial function [14, 17], and reducing ionic imbalance during ischemia and reperfusion [18]. Researchers have reported the protective effect of trimetazidine in CAD patients undergoing PCI [19, 20]. However, its long-term effect on left ventricular remodeling in CAD patients undergoing PCI is still unclear.

This prospective randomized trial is therefore designed to explore whether trimetazidine can improve ventricular remodeling in CAD patients with LVH, as well as trying to investigate whether trimetazidine treatment can reduce major adverse cardiovascular events (MACEs). This study is to provide evidence for further clinical application of trimetazidine in CAD patients undergoing PCI with LVH.

## Methods/design

### Study design

It is the first prospective, placebo-controlled study to explore the relationship between trimetazidine and ventricular remodeling. CAD patients undergoing PCI with LVH will be randomized to receive trimetazidine (35 mg twice a day) or placebo for 12 months after PCI. Coronary intervention and other medication therapy will be performed according to current guidelines, and no difference in the use of angiotensin-converting enzyme inhibitor (ACEI), angiotensin receptor blocker (ARB) and aldosterone antagonist should be ensured between the two groups. The web-based randomization will be used for a 1:1 ratio grouping to either trimetazidine or placebo. Secretary of department of cardiology accesses the website via account password and is responsible for randomization. Block randomization will be stratified by concomitant medications (ACEI, ARB or aldosterone antagonist), sex and family history and it is blind to those who enroll participants or assign interventions. Blood biochemistry, echocardiography, Seattle Angina Questionnaire, 6-min walk test (6-MWT), exercise electrocardiography test and MACEs will be collected at the 3- and 12-smonth clinical follow-ups. All personal information will be converted to the new subject number. The attending physicians are responsible for data entry. Data monitoring committee is responsible for data security and accuracy. Committee members (Fen Xu, Jinshan Tong and Jiangfen Jiang) have access to the final dataset and periodically range check for data values.

The primary end point is the change of ventricular remodeling assessed by left ventricular mass index (LVMI) at 3- and 12-month follow-up after PCI compared with the baseline. The secondary end points will be the symptom of angina assessed by Seattle Angina Questionnaire, myocardial ischemia measured by 6-MWT and exercise electrocardiography test, as well as MACEs (defined as a composite of death, myocardial infarction, stroke, recurrent angina, re-hospitalization, change of viable myocardium). Details of the proposed study, including enrollment and follow-up visits, are outlined in Table 1. All serious adverse events must be reported to the sponsor immediately or within 24 h in case report forms (CRFs).

**Table 1:** Study plan

Activity	Visit 0 screening	Visit 1	Visit 2
	0 day	3 months ±7 day	12 months ±7 day
Inclusion/exclusion criteria	×		
Informed consent	×		
Randomization	×		
Demography and medical history	×		
Physical examination	×	×	×
12-lead ECG	×	×	×
Concomitant medication	×	×	×
CBC	×	×	×
Scr, BUN, UA	×	×	×
ALT, AST	×	×	×
TC, LDL-C, HDL-C, TG	×	×	×
NT-proBNP	×	×	×
cTnT/cTnI	×		
Echocardiography	×	×	×
Symptoms of angina	×	×	×
6 min walk test	×	×	×
Exercise electrocardiography test	×	×	×
MACEs		×	×
Quality of life	×	×	×

*ECG* Electrocardiography, *CBC* complete blood count, *Scr* serum creatinine, *BUN* blood urea nitrogen, *UA* uric acid, *ALT* alanine aminotransferase, *AST* aspartate aminotransferase, *TC* total cholesterol, *LDL-C* low-density lipoprotein cholesterol, *HDL-C* how-density lipoprotein cholesterol, *TG* triglyceride, *NT-proBNP* N-terminal pro-B–type natriuretic peptide, *cTnT/cTnI* cardiac troponin-T/ cardiac troponin-I, *MACEs* major adverse cardiovascular events

The trial protocol has been approved by the Institutional Ethics Committee of Sir Run Run Shaw Hospital of Zhejiang University (KY20180508–25) and has been registered in at *Chinese Clinical Trial Registry* (ChiCTR1800017876). All patients will provide written informed consents before randomization.

### Study objectives

The trial has been designed to investigate the hypothesis that trimetazidine treatment (35 mg twice a day, for 12 months) could significantly improve the left ventricular remodeling, reduce the incidence of MACEs and effectively improve myocardial ischemia in CAD patients undergoing PCI with LVH. The primary objective is to validate that trimetazidine treatment could reduce LVMI and improve ventricular remodeling. The secondary objective is to validate that trimetazidine treatment could reduce the risk of MACEs, as well as improve the symptom of angina and myocardial ischemia.

### Inclusion and exclusion criteria

One hundred and twenty-four CAD patients undergoing PCI with LVH (LVMI > 120 and 100 g/m^2^ for men and women, respectively) at Sir Run Run Shaw Hospital of Zhejiang University will be enrolled in the trial. The enrollment period is expected to be finished in 2020. Detailed inclusion and exclusion criteria are listed in Table 2. The attending physician is responsible for the inclusion of patients. Patients treated with other medications which would affect cardiac metabolism, and patients who are concomitant with other causes of myocardial ischemia or other causes of LVH will be excluded from the study.

**Table 2:** Inclusion and exclusion criteria

**Inclusion Criteria**	
● CAD patients with hypertensive LVH who receive PCI.	
● Male and female aged 18–70 years.	
● Coronary stenosis is greater than 70% before PCI performed, and < 20% after PCI, with no need for other angioplasty.	
● Trimetazidine naive.	
● Provided informed consent.	
**Exclusion Criteria**	
● Utilization of trimetazidine, coenzyme Q10 or other medications which would affect cardiac metabolism.	
● Concomitant with other causes for left ventricular hypertrophy, such as aortic constriction, hypertrophic cardiomyopathy and cardiac valvular disease.	
● Concomitant with other causes for myocardial ischemia, such as congenital heart disease, rheumatic heart disease, dilated cardiomyopathy.	
● Presentation with an acute myocardial infarction.	
● Severe liver and kidney dysfunction (hepatic transaminase levels > 3 x ULN, bilirubin > 1.5 x ULN, eGFR < 30 mL/min/1.73m^2^).	
● Heart function defined as New York Heart Association IV, or LVEF < 30%.	
● Pregnancy or lactating, or possibility of a future pregnancy.	
● History of cancer.	
● Life expectancy less than 12 months.	

*CAD* coronary artery disease, *LVH* left ventricular hypertrophy, *PCI* percutaneous coronary intervention *ULN* upper limit of normal range, *eGFR* estimated glomerular filtration rate, *LVEF* left ventricular ejection fraction

### Exclusion criteria during the follow-up periods

Every participant will have the right to withdraw from the study without restriction, and participants may be removed if

1. Patients’ demand to withdraw the informed consent.
2. Study protocol violation (including poor compliance).
3. Lost to follow-up.
4. Pregnancy or new-onset malignant tumor during the follow-up.
5. Detrimental to the patient’s well-being.

Every withdrawal will be recorded in the CRFs, and a visit will be arranged if possible.

### Study procedures

**Fig. 1:**
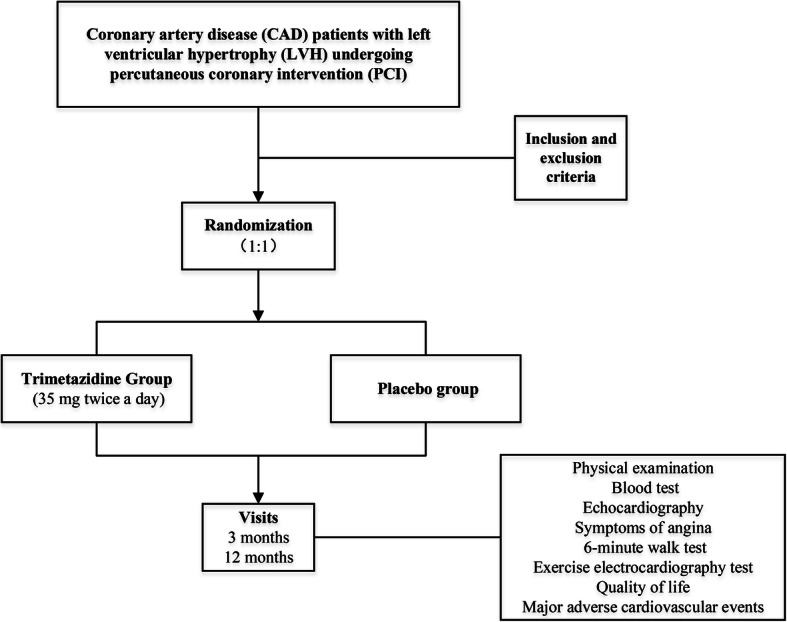
Flow chart

The study protocol is shown in Fig. 1 and Table 1. Patients are randomly allocated to receive trimetazidine (35 mg twice a day) or placebo for 12 months after PCI. All interventions are performed strictly comply with the standard. Physical examination, blood test, 12-lead electrocardiogram, echocardiography, symptoms of angina, 6-MWT, exercise electrocardiography test and quality of life will be collected at baseline visit and every follow-up visit. Blood tests will include complete blood count (CBC), alanine aminotransferase (ALT), aspartate aminotransferase (AST), serum creatinine (Scr), blood ureanitrogen (BUN), low-density lipoprotein cholesterol (LDL-C) and N-terminal pro-B–type natriuretic peptide (NT-proBNP). MACEs will be collected at 3- and 12-month follow-up. Research nurses will inform patients of the follow-up time in advance. They are helpful for completion of follow-up and monitoring adherence (e. g, drug tablet return).

### Primary end point

The primary end point in this study is the change between baseline and follow-up LVMI (calculated as the ratio: left ventricular mass (LVM)/ body surface area). Two-dimensional and Doppler echocardiography will be performed to quantify left ventricular function and structure (Phillips Healthcare) [21]. Ventricular remodeling will be assessed by calculating LVMI using Devereux formula. Interventricular septum thickness (IVST), left ventricular end-diastolic dimension (LVEDd), left ventricular posterior wall thickness (LVPWT) will be measured by echocardiography. LVM = 1.04[(IVST + LVEDd + LVPWT)^3^-LVEDd^3^]-13.6 g.

### Secondary end points

The secondary end points are the change the symptom of angina and myocardial ischemia, and the 3- and 12-month incidence of MACEs. The symptom of angina will be assessed at baseline, 3- and 12-month follow-up period by Seattle Angina Questionnaire. The improvement of myocardial ischemia and exercise capacity will be measured using exercise electrocardiography test and 6-MWT. The 6-MWT is a sample, low-cost method for estimating exercise capacity and will be performed according to standard procedures [22]. Clinical characteristics and a multipurpose health survey that consists of 36 questions (short form-36, SF-36) will be recorded at baseline, 3 months and 12 months. MACEs are defined as a composite end point of death, myocardial infarction, stroke, recurrent angina, re-hospitalization, change of viable myocardium.

### Trial committees

Wenbin Zhang guides the research team and provides overall supervision for the trial. The data monitoring committee (Fen Xu, Jinshan Tong and Jiangfen Jiang) is responsible for reviewing and monitoring data safety. Investigators have the right to reveal a participant’s allocated intervention or terminate the study in advance after discussion with Wenbin Zhang in the event of safety issues during the study as well as notify the ethics committee.

### Statistical considerations

#### Sample size

According to previous study [20], we hypothesized trimetazidine group has an effective rate of 70% to improve the left ventricular remodeling by reducing LVMI and placebo group has 40%, thus 56 patients in each group are necessary to offer 90% power at 0.05 significant level according to 1:1 ratio of random grouping. Anticipating 10% of patients potentially non-assessable according to previous experience on 12-month study, 124 randomized patients in total are needed.

#### Analysis

The final results will be presented on an intention-to-treat (ITT) basis. All data will be presented as percentages or mean ± standard deviation (SD). The comparison of data will be performed using the Chi-squared test (categorical variables) or Student’s t test (continuous variables). Cumulative incidences of end points in 12 months after PCI will be calculated using Kaplan-Meier methods. Log-rank tests will be used for comparisons between groups. Possible covariables will be incorporated in the analysis if certain important baseline levels are still not matched after randomization. Mean imputation will be used to handle missing data. A *p*-value < 0.05 will be considered significant. All statistical analyses will be performed using the Statistical Package for Social Sciences (SPSS), version 20.0 (SPSS Inc., Chicago, IL, USA). The results will be published by chief investigator and major contributors.

## Discussion

The study is the first investigator-initiated, prospective study to evaluate the treatment effect of trimetazidine on ventricular remodeling in CAD patients undergoing PCI with LVH. The result of the study will confirm whether trimetazidine improves ventricular remodeling, angina and myocardial ischemia.

Cardiac hypertrophy is a common pathological change in cardiovascular diseases. In the early stage, it is an adaptive response of the heart to the overload. With the development of the disease, the myocardial structure will be disordered and the compliance will be decreased. The mechanism of CAD associated with LVH is a complex process involving multiple factors, such as blood pressure, catecholamine and renin-angiotensin-aldosterone system. It is characterized by hypertrophy of cardiomyocytes and changes in coronary microcirculation. Coronary microcirculation networks are the metabolic site of myocardium, and their lesions can cause the decline of coronary reserve function and the changes of myocardial ultrastructure, which are closely related to the maintenance of myocardial energy metabolism.

Myocardial energy metabolism disorders occur prior to typical morphological changes in ventricular hypertrophy [23]. During the development of cardiac hypertrophy, the energy metabolism of hypertrophic cardiomyocytes changes from aerobic oxidation to anaerobic fermentation. Metabolic therapy is to improve the process of energy metabolism so that cardiomyocytes can get more energy. Trimetazidine is a metabolic agent with a protective effect on cardiomyocytes. It mainly inhibits the oxidation of free fatty acids, activates pyruvate dehydrogenase, increases the oxidation of glucose and the production efficiency of cardiac adenosine triphosphate (ATP), as well as maintains myocardial contractile function [24]. It also directs free fatty acids to synthesize phospholipids, which involve in the construction of cell membrane. Accordingly, by improving the environment of the cell and promoting the construction of membrane, trimetazidine might protect the ultrastructure of myocardium and is likely to inhibit or even reverse the cardiac hypertrophy.

Previous study has showed trimetazidine minimizes myocardial reperfusion injury during PCI and improves global and regional wall motion at one and 3 months after PCI [20]. In this previous study, 52 acute coronary syndrome (ACS) patients were included and randomized into trimetazidine group and placebo group. Echocardiographic measurements before revascularization revealed 40% patients in trimetazidine group had an ejection fraction < 50% versus 33% patients in placebo group. The number of patients with an ejection fraction < 50% was significantly reduced in trimetazidine group compared with placebo group after PCI (11% vs. 16%, *p* = 0.046 at 1 month; and 4% vs. 16%, *p* = 0.017 at 3 months). A significant improvement in regional wall motion was noted after treatment with trimetazidine compared with placebo. Three months after PCI, inferior left ventricular wall hypokinesia had improved in 66.7% trimetazidine recipients and in 42.9% placebo recipients (*p* = 0.05), and anterior wall hypokinesia had improved in 72.7% in trimetazidine group and in 33.3% in placebo group (*p* = 0.04). However, the researchers only focused on the improvements for 3 months, long-term impact of trimetazidine on left ventricular remodeling in CAD patients undergoing PCI is still unclear. Subsequent Korean study suggested that trimetazidine treatment in non-ST segment elevation myocardial infarction (NSTEMI) patients provided improvements in left ventricular end diastolic volume [25]. The adjuvant treatment with trimetazidine after drug-eluting stent implantation was also confirmed to have a beneficial effect on left ventricular function and structure in elderly multivessel CAD patients with diabetes mellitus [26]. Nonetheless, these studies were not focused directly on patients with LVH. The study we proposed is intended to provide evidence for further clinical application of trimetazidine in CAD patients with LVH.

There were also studies in recent years exploring possible molecular mechanisms. The rat model of aortic constriction was established to induce myocardial metabolic disorder within 7 days, and it was found that trimetazidine pretreatment could improve the myocardial energy metabolism by reducing serum levels of oxidative stress markers, attenuating the induction of genes related to myocardial hypertrophy, inhibiting the up-regulation of serum neuropeptide Y (NPY) levels, and further increasing the expression of myocardial NPY receptors [27]. This not only demonstrated that trimetazidine pretreatment could reverse acute metabolic disorders, but also revealed the underlying pathophysiological process of metabolic remodeling before the onset of myocardial remodeling.

Other researchers verified in vivo and in vitro that trimetazidine could effectively inhibit myocardial fibrosis through nicotinamide adenine dinucleotide phosphate (NADPH) oxidase-reactive oxygen species (ROS)-connective tissue growth factor (CTGF) signaling pathway [28]. However, those results were obtained just from animals and need to be supported by clinical trials.

In our study, stratified randomization will be used to reduce the possibility of variable imbalance. Thus, no difference will exist in the use of ACEI, ARB and aldosterone antagonist between groups. Nonetheless, it is possible that some baseline levels are still not matched after randomization. In this case, we believe that it occurs “by chance” and is a small probability event. Those variables (*p* < 0.05) will be adjusted. But in addition to statistical considerations, we will also assess the relationship between the adjusted covariates and the end points, rather than unselectively adjust them.

This study has some limitations. First, though the proposed study is designed as a randomized, placebo-controlled study, it is a single-center study, which may cause the baseline drift of the selected patients. Second, the causes of LVH are various. Hypertension, obesity and other pathologic disorders can cause an increase in the hemodynamic burden and lead to LVH. Thus, the findings of this study may not be applied to LVH caused by all diseases.

In conclusion, this trial will first investigate trimetazidine treatment (35 mg twice a day for 12 months) on the ventricular remodeling in CAD patients with LVH. We expect that the study results will confirm whether trimetazidine treatment can significantly improve ventricular remodeling.

## Data Availability

Not applicable.

## Abbreviations

CAD: Coronary artery disease
PCI: Percutaneous coronary intervention
LVH: Left ventricular hypertrophy
MACEs: Major adverse cardiovascular events
LVM: Left ventricular mass
LVMI: Left ventricular mass index
ChiCTR: Chinese clinical trial registry
ACEI: Angiotensin-converting enzyme inhibitor
ARB: Angiotensin receptor blocker
6-MWT: 6-min walk test
CRFs: Case report forms
CBC: Complete blood count
ALT: Alanine aminotransferase
AST: Aspartate aminotransferase
Scr: Serum creatinine
BUN: Blood urea nitrogen
LDL-C: Low-density lipoprotein cholesterol
NT-proBNP: N-terminal pro-B–type natriuretic peptide
IVST: Interventricular septum thickness
LVEDd: Left ventricular end-diastolic dimension
LVPWT: Left ventricular posterior wall thickness
SF-36: Short form-36
ITT: Intention-to-treat
SD: Standard deviation
SPSS: Statistical Package for Social Sciences
ATP: Adenosine triphosphate
ACS: Acute coronary syndrome
NSTEMI: Non-ST segment elevation myocardial infarction
NPY: Neuropeptide Y
NADPH: Nicotinamide adenine dinucleotide phosphate
ROS: Reactive oxygen species
CTGF: Connective tissue growth factor
ECG: Electrocardiography
UA: Uric acid
TC: Total cholesterol
HDL-C: How-density lipoprotein cholesterol
TG: Triglyceride
cTnT/cTnI: Cardiac troponin-T/ cardiac troponin-I
ULN: Upper limit of normal range
eGFR: Estimated glomerular filtration rate
LVEF: Left ventricular ejection fraction
